# Prescribing Hydroxyurea in Sickle Cell Disease Patients: The Pattern and Association with Co-Prescribed Medications Used to Manage the Disease Complications

**DOI:** 10.3390/jcm13206254

**Published:** 2024-10-19

**Authors:** Nida Alsaffar, Mohammed Ali Alsaleh, Abdulmonem Ali Alsaleh, Neda Z. Ghanem, Mohammad Hussain Al khamees, Mohammed A. Alqurain, Jenan Almatouq, Bader AlAlwan, Aymen A. Alqurain

**Affiliations:** 1 Department of Medical Laboratory Sciences, Mohammed Al-Mana College for Medical Sciences, Al Safa, Dammam 34222, Saudi Arabia; n.alsaffar@machs.edu.sa (N.A.); 2021101093@machs.edu.sa (M.A.A.);; 2 Blood and Cancer Research Department, King Abdullah International Medical Research Centre (KAIMRC), King Saud Bin Abdulaziz University for Health Sciences (KSAU-HS), Ministry of National Guard-Health Affairs (MNG-HA), Riyadh 11481, Saudi Arabia; 3 Department of Respiratory Therapy, Mohammed Al-Mana College for Medical Sciences, Dammam 34222, Saudi Arabia; n.ghanem@machs.edu.sa; 4 Department of Diagnostic Laboratory, King Fahad Hospital Hofuf, Ministry of Health, Al Hofuf 36441, Saudi Arabia; 5 Department of Diagnostic Laboratory, Maternity and Children Hospital, Ministry of Health, Al Mubarraz 36422, Saudi Arabia; 6 Department of Clinical Practice, Faculty of Pharmacy, Northern Border University, Rafha 91911, Saudi Arabia

**Keywords:** sickle cell disease, hydroxyurea, analgesics, adherence, pattern of use

## Abstract

**Background and Objectives:** Hydroxyurea (HU) is an effective medication used to reduce the frequency of painful crises associated with sickle cell disease (SCD). However, data describing its prevalence among SCD patients in the Eastern Region of Saudi Arabia are scarce. This is a multi-center, retrospective, cross-sectional study that aims to investigate the pattern of prescribing HU in SCD patients and to determine the association between prescribing HU and other co-prescribed medications used to manage SCD complications. **Methods:** Data were collected from patients who visited the hematology clinics of Al-Qatif Central Hospital (QCH) and King Fahad Hospital in Hofuf (KFHH) between June 2021 to May 2023. The data included demographics, prescribed medications, and recent laboratory test results, all of which were collected from patients’ medical records. Descriptive statistics were utilized to assess the difference between HU users vs. non-users. A binary logistic regression model was used to determine the association between prescribing HU and co-prescribed medications used to manage SCD complications. The results are presented as the odds ratio (OR) and 95% confidence interval (95% CI). **Results:** This study included 2816 SCD patients with a 56% prevalence of HU prescription. HU was prescribed for young age groups more often compared to old age group patients. Young males were more likely to be prescribed with HU compared to females, and it becomes dominant in females after the age of 36. HU users were more likely to have paracetamol (69% vs. 53%, OR = 1.9, 95% CI 1.6–2.2), NSAIDs (50% vs. 35%, OR = 1.7, 95% CI 1.5–2), and opioids (41% vs. 37%, OR = 1.3, 95% CI 1.1–1.6) co-prescribed, and less often to have laxatives (8% vs. 5%, OR = 0.66, 95% CI 0.48–0.9) and anticoagulants (22% vs. 15%, OR = 0.56, 95% CI 0.46–0.68) co-prescribed compared to non-users. **Conclusions:** The pattern of prescribing HU, supported by the association findings, raises concerns about patients’ compliance and adherence to HU therapy. Early health education, specifically to young female SCD patients, is warranted to increase the success rate of HU therapy.

## 1. Introduction

Sickle cell disease (SCD) is a serious inherited autosomal recessive hemoglobinopathy that affects millions of people worldwide [1,2,3]. SCD results from the development of hemoglobin S (HbS), which leads to the impairment of red blood cells’ (RBCs) function and, consequently, several clinical manifestations such as hemolytic anemia and vaso-occlusion that can affect several body organs [4,5]. SCD can have a significant financial, social, and psychosocial impact on individuals, families, and health systems [6,7].

Management options for SCD vary according to the clinical presentation and occurrence of acute or chronic complications. Analgesics, antibiotics, oxygenation, hydration, and blood transfusions are some management options for SCD [5]. Hydroxyurea (HU) is a well-known disease-modifying therapy that inhibits ribonucleotide reductase and increases hemoglobin F (HbF) in RBCs. HU subsequently decreases the expression of RBC adhesion molecules, white blood cell count, reticulocytes, and platelets [8].

HU was first introduced for SCD treatment in 1948 by Platt and her colleagues. It was approved by the Food and Drug Administration (FDA) in the United States to be used for adult SCD patients in 1998 and for children with SCD from two years and older in 2017 [9]. HU has been effective in reducing the frequency of painful episodes and acute chest syndrome in SCD patients, improving their quality of life and reducing their risk of death [10].

Although HU is considered a safe and effective drug for SCD management, discontinuation and resistance to use HU among SCD patients have been reported [5,11,12]. Several studies demonstrated the underutilization of HU among SCD patients due to different beliefs and attitudes [13]. A recent study reported that 57% of SCD patient discontinued their HU therapy as result of concerns for carcinogenesis and teratogenesis side effects [14,15]. Another study involving a sample of 107 SCD patients reported that less than 70% of the patients had heard of hydroxyurea and half of those who received the medication discontinued it as result of concerns toward HU long-term adverse drug reaction (ADR) [13].

While HU is an effective treatment for SCD, it is important to think about its potential side effects, especially for vulnerable populations like younger patients and pregnant women. Some of the common side effects involve myelosuppression, gastrointestinal disturbances, and dermatological reactions [16]. Concerns arise about the effect of HU on growth and development in younger patients, as well as potential fertility implications [17]. For pregnant women, the theoretical risk of teratogenic effects necessitates careful monitoring, especially since the discontinuation of HU can lead to increased pain crises, posing risks to both maternal and fetal health [18]. In order to optimize treatment strategies in these populations, it is crucial to have an understanding of HU’s side effects and long-term safety profile.

SCD is one of the most common blood inherited disorders in the Eastern Region of Saudi Arabia. According to the Saudi Ministry of Health, approximately 4.2% of the Saudi population have the sickle cell trait, and about 0.26% of the population have SCD. Specifically, SCD is most prevalent in the Eastern Province, representing about 17% of the population, with 1.2% having SCD [19]. However, clinical studies investigating patterns of prescribing HU are limited. Therefore, the aim of this study is to elucidate the pattern of prescribing HU as it is the approved treatment protocol for SCD in Saudi Arabia. This study will also highlight the association between prescribing HU and co-prescriptions of medications for managing SCD complications. To our knowledge, this study is the first study that demonstrates the trend of prescribing HU among different age groups stratified by gender and the intensity of HU dose. Also, it is the first to assess the likelihood of concurrent use of medications known to manage SCD complications and concurrent prescription of HU, which will help to determine how effective HU therapy is in managing SCD.

## 2. Methods

### 2.1. Study Population

This is a multi-center, retrospective, cross-sectional study that included SCD patients who attended hematology clinics at two main hospitals in the Eastern Provence, Qatif Central Hospital (QCH) and King Fahad Hospital in Hofuf (KFHH), during a period from June 2021 to May 2023. Individuals aged between 18 and 95 years, diagnosed with SCD, who attended a hematology clinic, and had a complete medical record were included. The exclusion criteria were individuals younger than 18 or older than 95 years, who attended clinics other than hematology, were not diagnosed with SCD, and had incomplete medical records.

### 2.2. Data Collection

Data were collected from patients’ medical records and include demographic data, co-prescribed medications, and recent medical laboratory test results. Body mass index (BMI) and creatinine clearance (CrCl) was calculated and recoded [20,21].

The co-prescribed medications, including long-term and short-term, were collected and then coded as per the Anatomical Therapeutic Chemical (ATC) classification system [22]. Analgesic drugs were coded as paracetamol (N02BE01), non-steroidal anti-inflammatory drugs (NSAIDs) (M01A), or opioids (N02A). Anticoagulants (B01AA-AF, excluding B01AC), antiplatelets (B01AC), laxatives (A06A), and vitamins and supplements (A11 and A12) were identified and recorded [22]. Antibiotics indicated for respiratory tract infection were identified as per the Hirschmann recommendation and then were recorded [23].

The US National Heart, Lung, and Blood Institute (NHLBI) suggests starting HU at a dose of 15 mg/kg/day, with a 5 mg/kg/day dose increase every two months up to a maximum of 35 mg/kg/day [24]. Considering that the average body weight for an adult ranges from 60 to 80 kg based on race difference, we used the average of these two values (70 kg) to calculate the cutoff point for HU dose intensity [21]. Therefore, the prescribed HU dose in the current study was classified into low vs. high dose. Low doses refer to a dose ≤1250 mg, whereas high doses refer to a dose >1250 mg. Patients were classified based on their age into eight different groups (18–25, 26–35, 36–45, 46–55, 56–65, 66–75, 76–85, 86–95 years) to help in assessing the difference in the pattern of hydroxyurea prescribing among these groups of patients.

### 2.3. Statistical Analysis

Demographic variables, laboratory test results, and co-prescribed medications were reported using the mean and standard deviation (SD) for continuous parametric variables, the median/interquartile range (IQR) for continuous non-parametric variables, and number/frequency for binary variables. For comparisons of continuous variables, Student’s *t*-test for parametric data or the Mann–Whitney *U* test for non-parametric data was used, whereas the Chi square test was used to compare the frequency of categorical variables between groups. Analysis of variance (ANOVA) test was used to identify trends or changes of different age groups. A binary logistic regression model was performed to compute the unadjusted and adjusted odds ratios (ORs) and 95% confidence interval (95% CI) to describe the association between prescribing HU and co-prescribed medications used to manage SCD complications. Furthermore, a multinominal logistic regression model was performed to determine the association of prescribing low and high doses with co-prescribed medications used to manage SCD complication in reference to no HU prescription. Covariates were included in the models if they reached a level of statistical significance at *p* < 0.05 in univariate analysis. Multicollinearity was tested with the variance inflation factor. Statistical analysis was performed using the SPSS statistical package version 26 (SPSS, Inc., Chicago, IL, USA), and *p* ≤ 0.05 was considered statistically significant.

## 3. Results

### 3.1. Characteristics of the Cohort

This study included 2816 patients with a 56% prevalence of HU prescription (Table 1). The data show that the median age was 37 years (IQR 22–61 years), the average BMI was 28 (SD ± 33), and the average CrCl value was 128 (±56) for the entire cohort. The univariate analysis revealed that HU users had a higher CrCl value (130 vs. 125, *p* = 0.001) compared to non-users (Table 1).

In relation to the commonly co-prescribed medications linked to SCD complications, the analysis revealed that folic acid and vitamin D3 were the most common co-prescribed supplements (66% and 52%, respectively, of the entire cohort) (Table 1). Interestingly, HU users were prescribed analgesic medications (paracetamol 69% vs. 53%, *p* < 0.001, NSAIDs 50% vs. 35%, *p* < 0.001, and opioids 41% vs. 37%, *p* < 0.001) more often compared to non-users (Table 1). HU non-users were prescribed antibiotics related to respiratory infections (11% vs. 5%, *p* < 0.001), anticoagulants (22% vs. 15%, *p* < 0.001), and laxatives (8% vs. 5%, *p* = 0.01) more commonly compared to HU users. HU users were more likely to be prescribed folic acid (76% vs. 52%, *p* < 0.001), vitamin D3 (61% vs. 40%, *p* < 0.001), and calcium (40% vs. 23%, *p* < 0.001) but less likely to have iron supplements (5% vs. 9%, *p* < 0.001) compared to non-users (Table 1).

Further investigations were conducted to assess the difference in laboratory test results between HU users vs. non-users. The univariate analysis revealed that HU users presented with higher international normalize ratio (INR) values (1.5 vs. 1.4, *p* = 0.02), lactate levels (7.8 vs. 7.4, *p* = 0.006), iron levels (16 vs. 14.8, *p* < 0.001), and TIBC values (86.5 vs. 80.3, *p* < 0.001) compared to non-users (Table 2). In contrast, HU non-users had a higher hematocrit (Hct) value (32.5 vs. 31.6, *p* = 0.001), creatinine level (68.6 vs. 66.2, *p* = 0.01), and urea level (4.7 vs. 4.3, *p* = 0.001) compared to HU users (Table 2).

### 3.2. Pattern of Hydroxyurea Prescription

**Figure 1:**
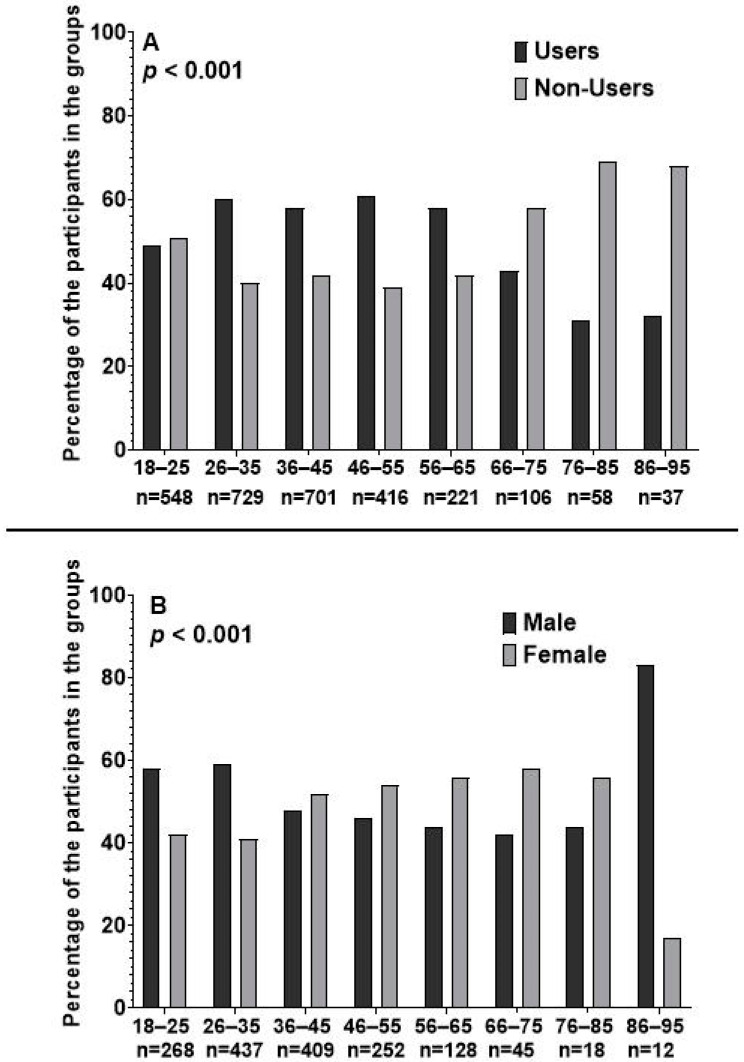
The trends of prescribing hydroxyurea. (**A**) Panel A represents the trend difference across different age groups. (**B**) Panel B represents the trend difference for each gender over the different age groups among hydroxyurea users.

A comparison between different age groups revealed that almost half of the patients aged between 18 and 25 years were prescribed HU (Figure 1). The prevalence of prescribing HU started to increase in response to the age increase, reaching its maximum value of 60% for patients in the age group of 56–65 years, and then, it began to decline (Figure 1). Furthermore, the analysis illustrated that within the early age groups, HU was prescribed to males more often than to females. The percentage of female users started to increase in response to the age increase, resulting in females being more dominant HU users than males in the age group of 36–45 years and above (Figure 1).

### 3.3. Total Prescribed Dose of Hydroxyurea

**Figure 2:**
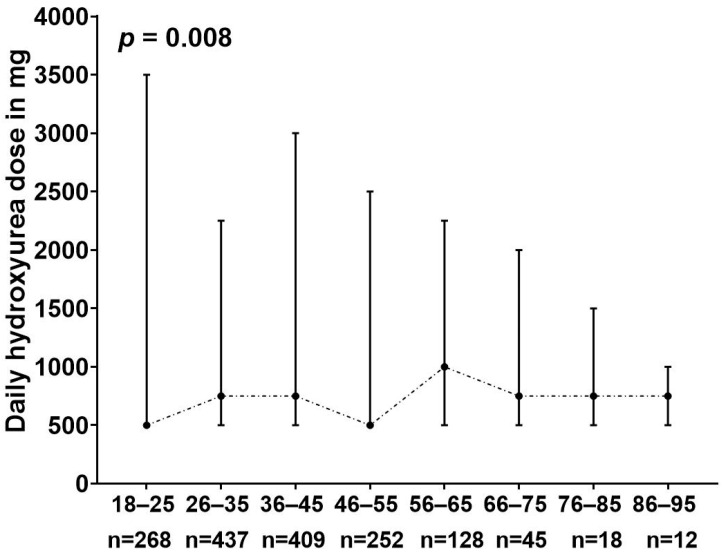
The daily prescribed hydroxyurea dose for sickle cell disease patients classified based on different age groups. The figure represents the median dose, the highest dose, and the lowest dose prescribed. *p* represents the *p* value generated from the ANOVA test.

Patients within the age group of 56–65 years were prescribed the highest median dose of HU compared to the other age groups (*p* = 0.008), whereas the highest reported daily dose of HU was within the age groups of 18–25 and 36–45 years (Figure 2).

### 3.4. High vs. Low Hydroxyurea Dose

The majority of the included patients were prescribed a low HU dose (91% of HU users), whereas high-dose users were older (37 vs. 42, *p* = 0.002) and more commonly prescribed folic acid (77% vs. 70%, *p* = 0.045) compared to those prescribed a low dose (Table 3).

**Figure 3:**
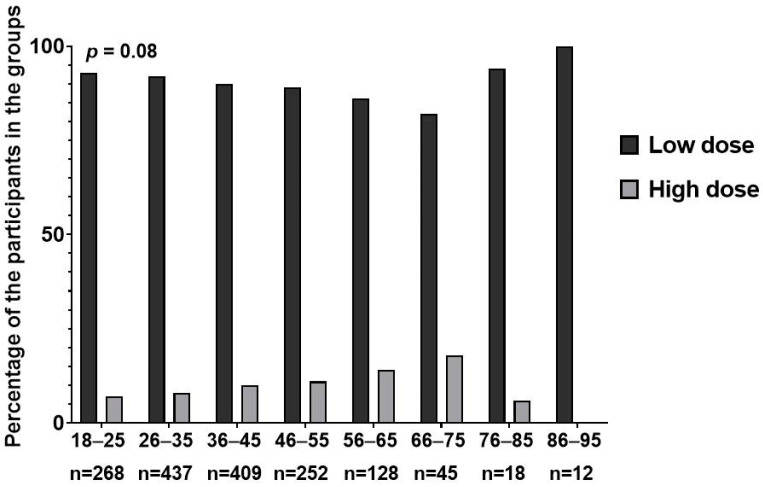
The differences in trends of prescribing high vs. low intensity doses of hydroxyurea across different age groups. *p* represents the *p* value generated from the ANOVA test.

Further analysis was conducted to assess the trends of prescribing high vs. low hydroxyurea doses, and the analysis revealed that prescribing low doses represented a downward curve, while high doses showed the opposite, an upward curve (Figure 3).

### 3.5. Association Between Prescribing Hydroxyurea and Co-Prescribing Medications Linked to SCD Complications

**Figure 4:**
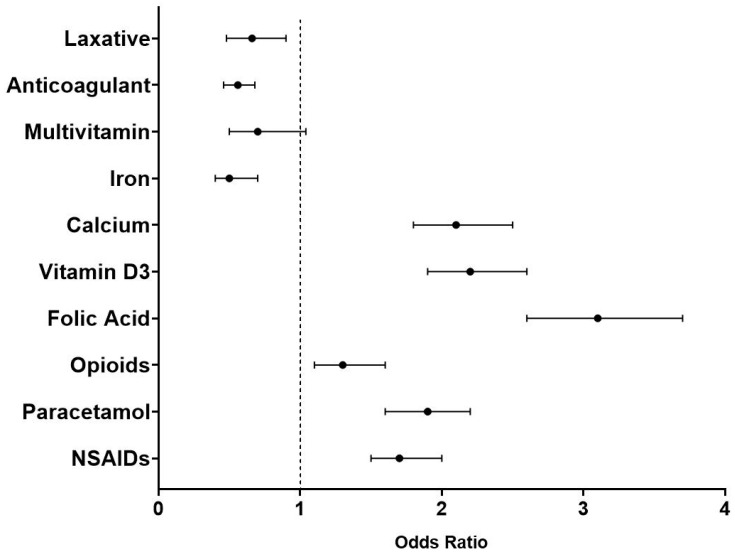
The association between prescribing hydroxyurea and co-prescribing medications linked to sickle cell disease complications. Odds ratio values were generated from the binary logistic model adjusted for age, gender, the body mass index value, and the creatinine clearance value.

A binary logistics regression model was conducted to determine the association with the overall trend of HU prescribing. The binary regression model was adjusted for age, gender, BMI, and CrCl. The analysis revealed that prescribing HU was associated with co-prescribing paracetamol (OR = 1.9, 95% CI 1.6–2.2), NSAIDs (OR = 1.7, 95% CI 1.5–2), opioids (OR = 1.3, 95% CI 1.1–1.6), folic acid (OR = 3.1, 95% CI 2.6–3.7), vitamin D3 (OR = 2.2, 95% CI 1.9–2.6), and calcium (OR = 2.1, 95% CI 1.8–2.5) (Figure 4). In contrast, a negative association between co-prescribing laxatives (OR = 0.66, 95% CI 0.48–0.9), anticoagulants (OR = 0.56, 95% CI 0.46–0.68), and iron (OR = 0.5, 95% CI 0.4–0.7) with HU prescribing was detected (Figure 4).

A multinominal regression analysis was performed to determine the association between low- and high-dose hydroxyurea prescribing and co-prescribing medications linked to SCD complications. The model was adjusted for age, gender, BMI, and CrCl (the OR value represents the likelihood in reference to no HU prescription). The analysis revealed that low-dose prescription was associated with co-prescribing paracetamol (OR = 1.87, 95% CI 1.59–2.21), NSAIDs (OR = 1.74, 95% CI 1.48–2.1), and opioids (OR = 1.29, 95% CI 1.09–1.52) (Table 4). Additionally, low-dose prescribing was associated with folic acid (OR = 3.2, 95% CI 2.68–3.82), vitamin D3 (OR = 2.17, 95% CI 1.85–2.55), and calcium supplement co-prescribing (OR = 2.15, 95% CI 1.81–2.56) (Table 4). Table 4 shows a negative association of co-prescribing multivitamins (OR = 0.69, 95% CI 0.49–0.989), anticoagulants (OR = 0.56, 95% CI 0.45–0.69), and laxatives (OR = 0.66, 95% CI 0.48–0.91) with prescribing low-dose HU (Table 4).

Similarly, high-dose prescribing was associated with co-prescribing paracetamol (OR = 1.78, 95% CI 1.2–2.6), NSAIDs (OR = 1.48, 95% CI 1.04–2.13), and opioids (OR = 1.7, 95% CI 1.16–2.37) (Table 4). Prescribing high-dose HU was associated with co-prescribing folic acid (OR = 2.5, 95% CI 1.73–3.8), vitamin D3 (OR = 2.37, 95% CI 1.66–3.4), and calcium (OR = 1.72, 95% CI 1.19–2.49) but negatively associated with iron supplements (OR = 0.25, 95% CI 0.09–0.7). Co-prescribing anticoagulants (OR = 0.54, 95% CI 0.34–0.87) was negatively associated with prescribing high-dose HU (Table 4).

## 4. Discussion

This study aimed to investigate the pattern of prescribing HU in SCD patients who attended a hematology clinic and to determine the association between prescribing HU and co-prescribing medications linked to managing SCD complications. This study found that the prevalence of prescribing HU was 56%. This value is considered within the range of the previously reported values (33–90%) from Saudi Arabia, but higher compared to another value reported from Oman (43%) [5,14,25]. This discrepancy might be due to the nature of the study design employed in the current study as it assesses the prevalence among SCD patients attending a hematology clinic, while others utilized a questionnaire or direct interview with the patients or their caregivers. The large sample size can be another reason for this discrepancy, as the current study involved 2816 patients, while the referenced studies reported a smaller sample size, varying between 107 and 1011 patients. This could strengthen the findings reported in this study as it assessed a large sample of patients based on their actual clinical context.

Another important finding from this study is the pattern of prescribing HU. This study reported that young age groups were prescribed HU more often compared to the old age groups. This finding sheds a light on the risk of HU underutilization, an issue reported in previous studies [26,27]. Several reasons could explain this pattern, such as a lack of awareness and poor compliance from patients and healthcare teams in relation to the concerns about fertility and the theoretical carcinogenic and teratogenic potential of HU [14,15]. In contradiction to this concern, HU therapy was not associated with an increased risk of cancer among SCD patients (overall cancer risk = 0.2%, 95% CI 0.0–0.3%) [16]. This reported risk value is not higher than the most recent published cancer incidence rate among the American population. Therefore, health education for SCD patients regarding the efficacy of HU should be put in practice to ensure patient adherence to HU therapy.

Another significant finding is that females were less likely to be prescribed HU in the younger age groups compared to males. This can be explained by patient willingness for conception and the desire to avoid the risk of teratogenicity associated with HU [15,18]. However, females with SCD experience severe pain, higher vaso-occlusive episodes, and have higher hospital admission rates compared to males [28]. For females with SCD and planning to become pregnant and seeking to discontinue HU, a comprehensive management plan is essential [18]. This plan should include frequent blood examinations and immediate access to emergency care for pain crises, along with strong communication with healthcare providers to ensure optimal monitoring and support throughout the pregnancy. This places emphasis on the importance of early health education with immediate consultation for those wishing to get pregnant and setting a care plan matching the patients’ needs.

It is important to note the disagreement between this study’s laboratory results and the literature regarding the effect of HU usage in SCD patients. A Brazilian study found that SCD HU users have a significant increase in HCT but insignificant changes in LDH, inflammatory markers, serum creatinine, and urea [29]. Additionally, another local study evaluated the laboratory markers for a cohort of SCD patients before and after HU usage and found insignificant changes in hemoglobin, HCT, and platelet count [30]. However, this inconsistency could be due to the confounding effect of age difference, as HU users in Yahouedehou et al.’s study were pediatric SCD patients [29]. Furthermore, gender differences could be another confounding factor as HU male users were found to have significantly higher hemoglobin, liver enzyme, serum creatinine, and urea levels than females [28].

Another important finding from this study is the association between prescribing HU and co-prescribing analgesic medications. Prescribing analgesics for SCD patients is warranted to manage the pain associated with SCD complications. such as vaso-occlusive episodes [31,32,33]. Such pain is expected to be controlled with ongoing HU use. This is supported by a recent finding from a study conducted in the Eastern Region of Saudi Arabia which showed that HU users have less pain and are less frequently hospitalized [34]. In the current study, the effectiveness of HU in managing the pain in the included cohort was not feasibly assessed due to being a retrospective study. Interestingly, HU users were prescribed more analgesic medications compared to non-users, which was supported by the regression finding as prescribing HU increases the likelihood of prescribing analgesics by 30–90% (based on the analgesic agent). One explanation for this pattern could be related to patient adherence to their HU therapy [34]. Due to fear of potential long-term toxicity, patient compliance and adherence to HU therapy is lower, which can be seen in this study as older patients or young female patients were less likely to have HU [32]. This conclusion supports the call for an early education intervention to ensure the highest adherence to the treatment plan.

In the current study, prescribing HU therapy varied significantly across different demographic groups. Notably, in young age groups, males were prescribed HU more often than females, but the ratio flipped among the old age groups, whereby females became dominant. As previously stated, young females may tend to refuse HU therapy to reduce its teratogenicity risk during pregnancy. On the other hand, one explanation for why females became dominant in using HU as they grew old is that females generally report their disease progression and seek healthcare advice for their health conditions more often compared to males [35,36]. This raises concerns about how effective the disease is managed among old male patients, questioning their compliance and adherence to HU therapy. Additionally, in the current study, young patients demonstrated a wider range of HU dose (500–3500 mg) compared to old patients (500–2500 mg), which raises concerns about how compliant and adherent young patients are to HU therapy. However, racial and ethnic data were not available to help in our understanding of how these variables affect compliance and adherence to HU therapy among the cohort. A previous published study reported that patients with low socio-economic status may exhibit a low compliance to HU therapy, which reflected the effectiveness of their disease management [37].

This is the first study to report the pattern of prescribing HU and examine the association with co-prescribing medications linked to managing SCD complications among SCD patients attending a hematology clinic in the Eastern Region of Saudi Arabia. Another strength of the current study is its substantial sample size and exploration of the difference between low and high doses of prescribing HU among gender or different age groups in the clinical setting.

However, several limitations should be acknowledged; as a retrospective cross-sectional study, it was difficult to accurately identify how effective HU therapy was in managing SCD owing to the inability to determine the exact initiation date of HU or the exact cause of using medications linked to SCD complications. Additionally, the cross-sectional design limited the ability to assess HU appropriateness and to interpret the ADRs broadly. The lack of follow-up data has also limited the understandings of longer-term treatment outcomes, including efficacy, ADRs, and readmission rates associated with HU use. The availability and accuracy of clinical data from medical records is another limitation to be considered. Missing data or inaccurate documentations are potential sources of information bias in this study. HU has been shown to be effective in reducing pain crises and improving overall outcomes in sickle cell disease patients. However, there is still a need for long-term data on its safety and efficacy among sicker patients. Another limitation of this study is the potential gap in understanding treatment variations across different centers and providers, which may influence prescribing patterns and patient outcomes. A comparative analysis of practices across various healthcare settings could provide valuable insights into how these differences impact the management of SCD. Lastly, due to its cross-sectional nature, this study did not allow for describing the trajectory of HU use over time and its associations with changes in the trajectories of SCD.

Based on our results, interventions should be implemented to enhance healthcare providers’ awareness of the potential risk of poor adherence and compliance to HU therapy, which contribute to exacerbating SCD complications. Health education campaigns on the safety and efficacy of HU should be tailored to young SCD patients, particularly females, to increase their awareness of the positive effect of HU on SCD management. Early diagnosis and proper treatment enable people with SCD to lead long, productive lives, necessitating ongoing access to quality healthcare, education, and support services. Future studies could benefit from including patient-reported outcomes to better understand the impact of HU on quality of life and pain management, which are crucial aspects of SCD management. Further research is required to focus on monitoring patients over extended periods to better understand any potential side effects or complications associated with long-term use. Future research should investigate the long-term safety profile of hydroxyurea, particularly for vulnerable populations like women of childbearing age or pediatric patients.

## 5. Conclusions

Within a cohort of SCD patients who attended a hematology clinic in the Eastern Region of Saudi Arabia, 56% of them were prescribed HU, with young age groups being prescribed HU more often compared to old age groups. Males were prescribed HU more often compared to females in young groups, but the inverse pattern was observed in old groups as females became dominant. Prescribing HU was associated with co-prescribing analgesic medications but negatively associated with anticoagulants and laxatives. The data generated from the current study raise concerns about the risk of HU underutilization as a result of less adherence or compliance to the therapy plan by patients. Therefore, early health education is crucial, particularly for female young patients, to improve HU therapy and ensure disease management.

## Figures and Tables

**Table 1:** Demographics and common co-prescribed medications for entire cohort classified based on hydroxyurea prescribing.

	Entire Cohort	HU Users	HU Non-Users	*p*-Value
	*n* = 2816	*n* = 1569 (56%)	*n* = 1247	
Age, median (IQR)	37 (22–61)	37 (23–58)	37 (21–66)	0.7
Gender (M), *n* (%)	1465 (52)	822 (52)	643 (52)	0.7
BMI, mean (±SD)	28 (±33)	28 (±33)	28 (±33)	0.5
CrCl, mean (±SD)	128 (±56)	130 (±53)	125 (±59)	0.001
Analgesics				
Paracetamol, *n* (%)	1733 (62)	1076 (69)	657 (53)	<0.001
NASIDs, *n* (%)	1220 (43)	779 (50)	441 (35)	<0.001
Opioids, *n* (%)	1122 (38)	650 (41)	462 (37)	<0.001
Supplements				
Folic acid, *n* (%)	1853 (66)	1199 (76)	654 (52)	<0.001
Vitamin D3, *n* (%)	1459 (52)	959 (61)	500 (40)	<0.001
Calcium, *n* (%)	904 (32)	623 (40)	281 (23)	<0.001
Iron, *n* (%)	184 (7)	73 (5)	111 (9)	<0.001
Multivitamins (B complex), *n* (%)	141 (5)	67 (4)	74 (6)	0.04
Others				
Antibiotics, *n* (%)	223 (8)	85 (5)	138 (11)	<0.001
Anticoagulants, *n* (%)	507 (18)	228 (15)	279 (22)	<0.001
Laxatives, *n* (%)	181 (6.4)	84 (5)	97 (8)	0.01

HU: hydroxyurea, BMI: body mass index, CrCl: creatinine clearance, IQR: interquartile range, M: male, and SD: standard deviation.

**Table 2:** The laboratory test results for the SCD patients classified based on HU prescribing.

	Entire Cohort	HU Users	HU Non-Users	*p*-Value
	*n* = 2816	*n* = 1569	*n* = 1247	
Complete blood count				
HGB, mean (±SD)	11 (2.6)	10.9 (2.6)	11 (2.6)	0.6
HCT, mean (±SD)	31.6 (8.6)	31 (8.3)	32.5 (8.9)	<0.001
Liver function test				
Albumin, mean (±SD)	40.8 (14.4)	40.7 (14.5)	41 (14.2)	0.4
ALT, mean (±SD)	56.3 (30.6)	56.5 (29)	56 (32.9)	0.8
AST, mean (±SD)	48.6 (24.3)	48.5 (20.5)	48.8 (29)	0.6
GGT, mean (±SD)	43.6 (20.2)	44 (19.9)	43 (20.6)	0.4
Bilirubin (conjugated), mean (±SD)	32.5 (22.2)	32.6 (22.4)	32.3 (21.9)	0.6
Bilirubin (total), mean (±SD)	38.8 (18)	38.3 (18.1)	39.4 (17.8)	0.06
INR, mean (±SD)	1.5 (0.5)	1.5 (0.6)	1.4 (0.5)	0.02
LDH, mean (±SD)	466.1 (367)	454.2 (344)	483.7 (399)	0.3
Lactate, mean (±SD)	7.6 (4.6)	7.8 (4.6)	7.4 (4.7)	0.006
Renal function tests				
Creatinine, mean (±SD)	67.2 (42)	66.2 (43)	68.6 (40.3)	0.006
Urea, mean (±SD)	4.5 (3.6)	4.3 (3.2)	4.7 (3.9)	<0.001
Iron studies				
Iron, mean (±SD)	15.5 (7.6)	16 (7.6)	14.8 (7.6)	<0.001
TIBC, mean (±SD)	84 (48.5)	86.5 (49.1)	80.3 (47.5)	<0.001
Ferritin, mean (±SD)	400.8 (1150)	427.5 (1303)	361.3 (874)	0.002
Other tests				
ESR, mean (±SD)	27.3 (18.3)	26.6 (18)	28.4 (18.8)	0.001
Folate, mean (±SD)	24.9 (10.4)	24.6 (10.5)	25.3 (10.2)	0.04
Vitamin D, mean (±SD)	83.6 (32.3)	84.1 (31.1)	82.8 (34.1)	0.6
Vitamin B12, mean (±SD)	388.4 (260.2)	385.8 (199.1)	392.2 (330.7)	0.5
Uric acid, mean (±SD)	326.4 (134.9)	325.8 (134.9)	327.3 (135.1)	0.8

HU: hydroxyurea, ALT: alanine transaminase, AST: aspartate aminotransferase, ESR: erythrocyte sedimentation rate, GGT: gamma-glutamyl transferase, HCT: hematocrit, HGB: hemoglobin, TIBC: Total iron binding capacity, INR: international normalized ratio, LDH: lactate dehydrogenase, and SD: standard deviation.

**Table 3:** Characteristics of hydroxyurea users classified based on hydroxyurea dose intensity.

	HU Users	Low Dose	High Dose	*p*-Value
	*n* = 1569	*n* = 1420	*n* = 149	
Age, median (IQR)	37 (23–58)	37 (23–57)	42 (25–62)	0.002
Gender (M), *n* (%)	822 (52)	743 (52)	79 (53)	0.8
BMI, mean (±SD)	28 (±33)	27.5 (±31)	30.6 (±52)	0.4
CrCl, mean (±SD)	130 (±53)	130 (±52)	129 (±60)	0.3
Analgesics				
Paracetamol, *n* (%)	1076 (69)	977 (69)	99 (66)	0.6
NASIDs, *n* (%)	779 (50)	713 (50)	66 (44)	0.2
Opioids, *n* (%)	650 (41)	582 (41)	68 (46)	0.3
Supplements				
Folic acid, *n* (%)	1199 (76)	1095 (77)	104 (70)	0.045
Vitamin D3, *n* (%)	959 (61)	867 (61)	92 (62)	0.9
Calcium, *n* (%)	623 (40)	572 (40)	51 (34)	0.2
Iron, *n* (%)	73 (5)	69 (5)	4 (3)	0.2
Multivitamins (B complex), *n* (%)	67 (4)	59 (4)	8 (5)	0.5
Others				
Antibiotics, *n* (%)	85 (5)	75 (5)	10 (7)	0.5
Anticoagulants, *n* (%)	228 (15)	204 (14)	24 (16)	0.6
Laxatives, *n* (%)	84 (5)	75 (5)	9 (6)	0.7

HU: hydroxyurea, BMI: body mass index, CrCl: creatinine clearance, IQR: interquartile range, M: male, and SD: standard deviation.

**Table 4:** Association between low vs. high dose of hydroxyurea prescription and co-prescribed medications linked to sickle cell disease complications.

Characteristic	Low Intensity Dose		High Intensity Dose	
	Odds Ratio	95% CI	Odds Ratio	95% CI
NSAIDs	1.74	1.48–2.1	1.48	1.04–2.13
Paracetamol	1.87	1.59–2.21	1.78	1.2–2.6
Opioids	1.29	1.09–1.52	1.7	1.16–2.37
Folic acid	3.2	2.68–3.82	2.5	1.73–3.8
Vitamin D3	2.17	1.85–2.55	2.37	1.66–3.4
Calcium	2.15	1.81–2.56	1.72	1.19–2.49
Iron	0.5	0.37–0.69	0.25	0.09–0.7
Multivitamins	0.69	0.49–0.989	0.8	0.37–1.7
Anticoagulants	0.56	0.45–0.69	0.54	0.34–0.87
Laxatives	0.66	0.48–0.91	0.64	0.31–1.31

Odds ratio values were generated from the multinominal logistic model adjusted for age, gender, the body mass index value, and the creatinine clearance value with reference to not prescribing hydroxyurea.

## Data Availability

The data that support the findings of this study are available from the corresponding author upon reasonable request.
